# Molecular Genetic Basis of Lab- and Field-Selected Bt Resistance in Pink Bollworm

**DOI:** 10.3390/insects14020201

**Published:** 2023-02-17

**Authors:** Jeffrey A. Fabrick, Xianchun Li, Yves Carrière, Bruce E. Tabashnik

**Affiliations:** 1 USDA ARS, U.S. Arid Land Agricultural Research Center, Maricopa, AZ 85138, USA; 2 Department of Entomology, University of Arizona, Tucson, AZ 85721, USA

**Keywords:** *Pectinophora gossypiella*, transgenic cotton, genetically engineered crop, *Bacillus thuringiensis*, resistance, cadherin, ATP-binding cassette transporter

## Abstract

**Simple Summary:**

The pink bollworm, *Pectinophora gossypiella*, is one of the most damaging pests of cotton worldwide. Cotton has been genetically engineered to produce insect-killing proteins from the bacterium *Bacillus thuringiensis* (Bt) to control major lepidopteran pests, including the pink bollworm. The Bt proteins in genetically engineered crops are not toxic to people, other vertebrates, or most beneficial insects. Advantages of Bt crops can include pest suppression, improved yields, increased farmer profits, and decreased use of conventional insecticides. In the United States, Bt cotton, sterile moth releases, and other tactics were used to eradicate the pink bollworm. For more than 20 years, Bt cotton has been effective against pink bollworm in China. However, the benefits of Bt crops are reduced when pests evolve resistance, as exemplified by pink bollworm resistance to Bt cotton in India. For each of the two Bt proteins used widely in Bt cotton, the genetic basis of resistance is similar between resistance selected in the lab versus the field, regardless of the country of origin. The results suggest that lab selection can be useful for identifying genes likely to be important in field-evolved resistance to Bt crops and that differences in management practices among countries caused different outcomes.

**Abstract:**

Transgenic crops producing insecticidal proteins from the bacterium *Bacillus thuringiensis* (Bt) control some important insect pests. However, evolution of resistance by pests reduces the efficacy of Bt crops. Here we review resistance to Bt cotton in the pink bollworm, *Pectinophora gossypiella*, one of the world’s most damaging pests of cotton. Field outcomes with Bt cotton and pink bollworm during the past quarter century differ markedly among the world’s top three cotton-producing countries: practical resistance in India, sustained susceptibility in China, and eradication of this invasive lepidopteran pest from the United States achieved with Bt cotton and other tactics. We compared the molecular genetic basis of pink bollworm resistance between lab-selected strains from the U.S. and China and field-selected populations from India for two Bt proteins (Cry1Ac and Cry2Ab) produced in widely adopted Bt cotton. Both lab- and field-selected resistance are associated with mutations affecting the cadherin protein PgCad1 for Cry1Ac and the ATP-binding cassette transporter protein PgABCA2 for Cry2Ab. The results imply lab selection is useful for identifying genes important in field-evolved resistance to Bt crops, but not necessarily the specific mutations in those genes. The results also suggest that differences in management practices, rather than genetic constraints, caused the strikingly different outcomes among countries.

## 1. Introduction

Crystalline (Cry) proteins from the bacterium *Bacillus thuringiensis* (Bt) kill some key insect pests yet are not toxic to people and most other non-target organisms [1,2,3,4,5]. Crops genetically engineered to produce Cry toxins include corn, cotton, cowpea, eggplant, soybean, and sugarcane [6,7,8,9]. These transgenic Bt crops can improve pest control, enhance yields, increase farmer profits, and decrease use of conventional insecticides, thereby benefiting human health and the environment [2,4,10,11,12,13]. However, evolution of pest resistance to Bt crops has reduced these benefits. Practical resistance to Cry toxins in Bt crops, which is field-evolved resistance that has practical consequences for pest management, has been documented in at least 26 cases involving 11 pest species in seven countries [14].

Understanding the genetic basis of resistance can be useful for monitoring, managing, and countering pest resistance to Bt crops [15,16,17,18]. To facilitate development of effective resistance management strategies, scientists have determined the genetic basis of resistance to Bt toxins in many lab-selected strains, including strains derived from susceptible populations by either mass selection or selection of families generated for F_1_ or F_2_ screens [19,20,21,22,23,24,25,26]. If lab-selected resistance and field-selected practical resistance have a similar genetic basis, the lab results can be useful for designing and proactively implementing strategies to delay resistance in the field [27]. Although gene editing with CRISPR/Cas9 has been effective for identifying genes in which mutations can cause resistance to Bt toxins [17,28,29,30,31,32,33,34,35,36,37], this approach cannot determine which genes are actually important in conferring field-selected practical resistance. As far as we know, the data required for comparing the molecular genetic basis between lab-selected resistance and practical resistance to a Bt crop for the same pest are available only for the pink bollworm (*Pectinophora gossypiella*), one of the world’s most damaging pests of cotton [38,39]. This review summarizes and synthesizes evidence from a quarter century of research that enables comparisons of the genetic basis of lab-selected resistance versus field-selected practical resistance to Bt toxins Cry1Ac and Cry2Ab in the pink bollworm.

The pink bollworm, an invasive lepidopteran species thought to have originated in India, Pakistan, Indonesia, or Australia, occurs across most of the tropics and sub-tropics in cotton-growing areas worldwide [39]. The larvae feed primarily on plants in the Malvaceae family, with a preference for seeds within cotton bolls of *Gossypium* species. Larvae are difficult to control inside cotton bolls, where they avoid exposure to insecticide sprays and attack by natural enemies. However, transgenic Bt cotton targets pink bollworm larvae within bolls, as the insecticidal Bt proteins are produced in both maternal plant tissues and seeds [40]. In 2019, farmers in 18 countries planted 25 million hectares of Bt cotton, which was 77% of all cotton planted worldwide [6]. Bt cotton producing Cry1Ac alone and Cry1Ac + Cry2Ab have been used widely for decades to control pink bollworm and other lepidopteran pests. Although some Bt cotton also produces Cry1Fa and/or Vip3Aa targeting several lepidopteran pests, these proteins are not highly effective against pink bollworm [41,42].

Below, we summarize the remarkably different field outcomes with pink bollworm and Bt cotton in the three countries that lead the world in cotton production: the United States, China, and India. Next, we briefly describe the mode of action of Cry toxins, emphasizing information relevant to pink bollworm. The remaining sections compare the genetic basis of pink bollworm resistance to Cry1Ac and Cry2Ab in lab-selected strains from the U.S. and China versus field-selected populations in India.

**Figure 1:**
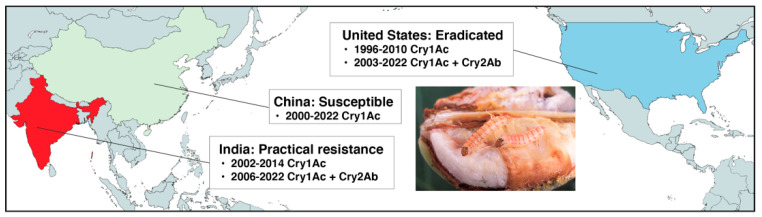
Pink bollworm responses to Bt cotton in the U.S., China, and India. Sustained susceptibility to Bt cotton producing either Cry1Ac or Cry1Ac + Cry2Ab was essential for the success of a multi-tactic program that eradicated pink bollworm from the U.S. [13]. In China, a small increase in resistance to Cry1Ac detected during 2008–2010 was reversed following extensive planting of F_2_ hybrid cotton produced by crossing non-Bt cotton with Bt cotton [44]. Pink bollworm evolved practical resistance to Bt cotton producing Cry1Ac and Cry1Ac + Cry2Ab in India, where refuges of non-Bt cotton were scarce [45].

As described below, pink bollworm evolved practical resistance to Bt cotton in India but not in the U.S. or China (Figure 1) [14,43]. We consider the hypothesis that relative to India, limited genetic variation of pink bollworm delayed its evolution of resistance to Bt cotton in the U.S. and China. The rationale is that India is apparently part of or close to the ancestral region of pink bollworm, whereas it more recently invaded the U.S. and China [39]. However, the results show a similar genetic basis of resistance among countries, which implies that differences in management practices rather than genetic constraints caused dramatically different outcomes among countries.

## 2. Field Outcomes: Pink Bollworm and Bt Cotton in the U.S., China, and India

The global monitoring data for pink bollworm and Bt cotton show sustained susceptibility followed by eradication of pink bollworm from the cotton-growing areas of the continental U.S. and northern Mexico, a small increase in resistance followed by restoration of susceptibility to Cry1Ac in China, practical resistance to Cry1Ac and Cry2Ab in India, and practical resistance to Cry1Ac in Pakistan [14,46]. We focus on the U.S., China, and India (Figure 1), which are the only three countries for which detailed information about the genetic basis of Bt resistance in pink bollworm is available.

### 2.1. Sustained Susceptibilty to Bt Cotton and Eradication of Pink Bollworm in the U.S.

In the southwestern U.S., including the state of Arizona, the pink bollworm was a major pest for much of the past century after its initial detection in 1917 [13]. In Arizona from 1996 to 2005, most growers complied with mandated planting of non-Bt cotton refuges and non-Bt cotton accounted for more than 25% of total cotton hectares statewide each year [46,47,48,49]. Various analyses support the conclusion that, together with fitness costs in the absence of Bt toxins associated with resistance and incomplete resistance [50,51,52], these refuges helped sustain pink bollworm susceptibility to Cry1Ac and Cry2Ab [47,48,49,53,54,55].

As part of the regional eradication program that included mass releases of sterile pink bollworm moths and other tactics [48,56,57], the U.S. Environmental Protection Agency allowed farmers in Arizona to forgo planting of non-Bt cotton refuges starting in 2006 [13,48]. The program began in parts of Texas, New Mexico, and northern Mexico in 2001 [56], then expanded west into Arizona and California. Its success led to the 2018 declaration by the U.S. Department of Agriculture that the pink bollworm was eradicated from the cotton-growing areas of the continental U.S. [13,58]. Sustained efficacy of Bt cotton producing Cry1Ac and Cry1Ac + Cry2Ab was a key component of the successful outcome [13].

### 2.2. Pink Bollworm Susceptibility to Bt Cotton Producing Cry1Ac Restored in China

In China, where pink bollworm is a pest in nearly all cotton-growing areas, this insect was first reported in 1918 [59]. Since 2000, millions of small-scale farmers have grown Cry1Ac-producing Bt cotton in the Yangtze River Valley of China, where pink bollworm is a primary pest [44]. Unlike the U.S. and other countries, China has not mandated the planting of non-Bt cotton refuges and has not approved planting of transgenic cotton that produces two or more Bt toxins targeting lepidopteran pests. In the Yangtze River Valley, non-Bt cotton accounted for an annual mean of 13% of all cotton hectares planted from 2007 to 2009 [44].

From 2008 to 2010, lab bioassays detected a small yet significant increase in resistance to Cry1Ac in pink bollworm strains derived from field populations in the Yangtze River Valley [60]. These data suggested that without major changes, resistance might quickly increase and have practical consequences. However, from 2010 to 2015, the percentage of cotton fields planted with seeds from F_2_ hybrids resulting from crosses between Bt and non-Bt cotton increased dramatically [44]. Sowing of such F_2_ hybrid seeds is expected to produce fields containing a random mix of approximately 25% non-Bt cotton plants and 75% Bt cotton plants. Results of modeling show that together with fitness costs associated with resistance and incomplete resistance, the increased planting of F_2_ hybrids is sufficient to account for the restoration of susceptibility to Cry1Ac that was seen in bioassays conducted from 2011 to 2015 [44]. Additional monitoring discussed in more detail below (Section 4.2.1) shows susceptibility to Cry1Ac was maintained at least through 2017 [61,62]. The voluntary planting of F_2_ hybrids apparently has immediate benefits in terms of lower seed costs and higher yields, as well as serendipitous benefits for managing resistance [44].

### 2.3. Practical Resistance of Pink Bollworm to Bt Cotton in India

The first description of pink bollworm is from India in 1843 [39]. Legal planting of Bt cotton producing Cry1Ac began in India in 2002 and was reportedly preceded by several years of illegal planting in western India [63,64]. Based on results from lab bioassays, field-evolved resistance of pink bollworm to Cry1Ac was first discovered in a strain derived in 2008 from a population in the western state of Gujarat [65]. By 2010, resistance to Bt cotton producing Cry1Ac was also found in the neighboring states of Maharashtra and Madhya Pradesh [66,67]. Bt cotton producing Cry1Ac + Cry2Ab was introduced in 2006, and widespread practical resistance to this two-toxin Bt cotton was documented in 2015 [45]. The rapid evolution of pink bollworm resistance to Bt cotton in India, which has caused substantial socio-economic damage, is associated with the failure to comply with the governmental requirement to plant non-Bt cotton refuges accounting for at least 20% of total cotton hectares [43,68,69,70,71]. Pink bollworm resistance to Bt cotton remained widespread in India through at least 2018, with no immediate prospects for restoration of susceptibility to Cry1Ac or Cry2Ab [43,45,72,73]. In 2019, 94% of all cotton planted in India was Bt cotton [6]. In the wake of pink bollworm resistance, the benefits of Bt cotton in India have been debated [74,75]. Factors favoring its continued high adoption may include limited availability of non-Bt cotton and the lack of practical resistance to Bt cotton in the major pest *Helicoverpa armigera* [76]. In India, abundant refuges of non-Bt host plants other than cotton may have helped to delay the evolution of practical resistance in this polyphagous pest [76].

## 3. Cry Toxin Mode of Action

**Figure 2:**
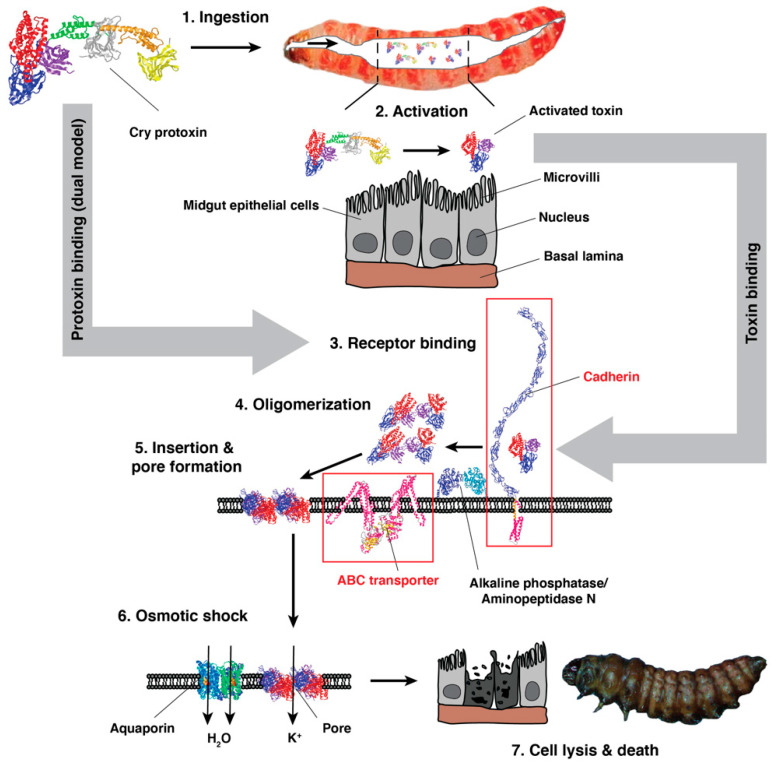
Cry toxin mode of action. Cry toxins must be ingested to kill susceptible larvae (Step 1). In the standard pore formation model, Cry protoxin must be activated by insect proteases (Step 2), and the smaller activated toxin binds to primary receptor proteins (e.g., cadherin and ABC transporters) at the surface of the midgut epithelium (Step 3). Next, Cry toxin oligomerizes (Step 4) and interacts with co-receptors (alkaline phosphatase and/or aminopeptidase N). The oligomers insert into the epithelial membrane forming pores (Step 5). The rapid influx of cations through these pores causes osmotic shock in the midgut cells (Step 6). The cells swell due to the uptake of water through aquaporin water channel proteins and eventually lyse. Ultimately, the insect dies from acute damage, starvation, and/or septicemia (Step 7). A new “dual” model proposes that protoxin, as well as activated toxin, binds to midgut receptors and causes toxicity via a second pathway.

To kill insects, Cry toxins must be ingested by larvae and bind to midgut receptor proteins such as cadherins and ABC transporters (Figure 2, Steps 1 and 3) [24,77,78,79,80]. For pink bollworm, reduced binding of Bt toxin to midgut receptors is the primary mechanism of resistance to Cry1Ac and is suspected to be the mechanism of resistance to Cry2Ab (see Section 4 below).

Whereas bacterial cells produce Bt proteins in crystals that require solubilization in the alkaline larval midgut, Bt plants produce soluble Bt proteins. Bt cotton producing Cry1Ac and Cry2Ab make the full-length protoxin form of these proteins (130 and 63 kDa, respectively) [1,81]. According to standard mode of action models, including the pore formation model, Cry protoxins must be converted by larval midgut proteases to activated toxins that bind to midgut receptors (Figure 2, Step 2) [79,80]. This step yields activated toxins of 65 kDa for Cry1Ac and 50 kDa for Cry2Ab [82,83]. However, Cry1Ac protoxin as well as activated toxin bind to recombinant fragments of the pink bollworm cadherin protein PgCad1 (formerly called BtR) [84]. This finding and subsequent results with several lepidopterans suggest that Cry1A protoxins and activated toxins kill larvae via different pathways (Figure 2, dual model) [82,85,86,87,88,89]. As far as we know, this hypothesis remains to be tested for Cry2A proteins.

In the widely accepted pore formation model of the Bt toxin mode of action, binding to midgut receptors is followed by the generation of toxin oligomers that insert into columnar epithelial cells (enterocytes) to create pores (Figure 2, Steps 4 and 5). These pores cause unregulated influx of cations into cells followed by water (via intrinsic aquaporin water channel proteins), which leads to swelling and lysis of cells (Figure 2, Step 6) [80,90]. Subsequently, the larva dies from acute damage to the cells of the midgut epithelium, starvation, and/or septicemia (Figure 2, Step 7). In the alternative signal transduction model (which is not depicted in Figure 2), the initial steps are the same as in the pore formation model but binding to cadherin activates an intracellular signaling pathway that causes oncocytic cell death [91,92]. Whereas the signal transduction model is based on results only from insect cell cultures, the extensive evidence supporting the pore formation model includes data from larvae of pink bollworm and other lepidopterans [15,24,80,93,94].

## 4. Pink Bollworm Resistance to Cry1Ac

### 4.1. Shared Mode of Resistance to Cry1Ac in the U.S., China, and India

The genetic basis of pink bollworm resistance to Cry1Ac is similar for lab-selected strains from the U.S. and China and field-selected populations from India (Table 1). In nearly all strains analyzed from these three countries, pink bollworm resistance to Cry1Ac fits “Mode 1” resistance, which was recognized early on as the most common type of resistance to Cry1A toxins in Lepidoptera [95]. In this case, Mode 1 resistance entails high levels of resistance to Cry1Ac, recessive inheritance, a narrow spectrum of cross-resistance to other Cry toxins, and reduced binding of at least one Cry1A toxin to larval midgut membranes [95].

The resistance ratio is the concentration of toxin killing 50% of larvae (LC_50_) for a potentially resistant strain divided by the LC_50_ for a susceptible strain. Resistance ratios for Cry1Ac exceeded 200 in lab-selected strains from the U.S. and China [61,96,97,98,99,100,101] as well as in the NKJ, IG09RP, and Jalagon-R strains from India, which were derived from field-selected populations and further selected with Cry1Ac in the lab [66,102,103]. Larval survival on Bt cotton producing Cry1Ac is generally increased for pink bollworm with such high resistance ratios relative to the survival at or close to 0% for susceptible pink bollworm [44,66,104].

Inheritance of resistance to Cry1Ac was autosomal in all the many strains of pink bollworm tested [61,98,99,100,101,102,103,104,105,106,107,108,109]. The dominance of resistance can be gauged using the parameter *h*, for which 0 indicates completely recessive resistance and 1 indicates completely dominant resistance [110]. It is most relevant for assessment of field-evolved resistance to estimate *h* from survival of neonates in bioassays at a diagnostic concentration of Cry1Ac (usually 10 micrograms Cry1Ac per ml diet) or on Bt cotton bolls producing Cry1Ac. In 17 such bioassays testing nine lab-selected strains from the U.S. and China, *h* was 0, indicating recessive inheritance of resistance [44,51,61,98,99,100,101,102,103,105,106,107,108,109,111].

Similar to the results from the U.S. and China, Nair et al. (2016) [102] found that resistance in the NKJ strain from India was completely recessive at concentrations at or above 1.0 microgram Cry1Ac per ml diet. However, based on LC_50_ values that are not necessarily relevant to the field, *h* was 0.22 for NKJ [102]. Also based on LC_50_ values, Mittal et al. (2016) [103] estimated *h* was 0.83–0.84 for the Jalagon-R strain from India. They did not report results from tests at single toxin concentrations. The anomalously high values of *h* were determined by testing F_1_ progeny from crosses between Jalagon-R and an unrelated susceptible strain, but the results from some subsequent crosses are consistent with recessive inheritance. For example, the LC_50_ of Cry1Ac did not differ significantly between the susceptible strain and the pooled progeny from the F_1_ (Jalagon-R X susceptible) backcrossed to the susceptible strain [103]. Whereas other studies tested neonates, five-day-old larvae were tested from Jalagon-R, which could have contributed to the overestimation of *h* for Jalagon-R in two ways: (1) Pink bollworm neonates are exposed to Bt toxins in Bt cotton and are more susceptible to Cry1Ac than older larvae [112]. So, for any given concentration of Cry1Ac, five-day-old larvae are expected to have higher survival than neonates. Because dominance of pink bollworm resistance to Bt toxins increases as survival increases [107,113], *h* is expected to be higher for five-day-old larvae than neonates. (2) During the five days while larvae were reared on an untreated diet before bioassays, hybrid vigor resulting from crossing Jalagon-R with an unrelated susceptible strain could have yielded faster development of the F_1_ progeny than the parental strains. This would increase the survival of the F_1_ progeny relative to the parental strains and thereby overestimate *h.*

Pink bollworm strains from all three countries that were selected with Cry1Ac in the lab, field, or both had little or no cross-resistance to Cry2Ab [41,61,65,66,99,100,101,107,114,115,116]. The resistance ratio for Cry2Ab was less than three in all nine bioassays testing seven Cry1Ac-selected strains from the U.S., China, and India (mean = 1.7, SE = 0.2) [61,99,100,101,102,115,116]. These results are consistent with the finding that binding sites are not shared by Cry1Ac and Cry2Aa (which is closely related to Cry2Ab) in pink bollworm [117].

Relative to susceptible strains, binding of Cry1Ac to larval midgut brush border membrane preparations was reduced in the NKJ strain and field-selected populations from India [67] but not in the lab-selected AZP-R strain from the U.S. that had up to 3100-fold resistance to Cry1Ac [94,107,118]. However, AZP-R did show reduced binding of the closely related toxin Cry1Ab [118] and reduced oligomerization of Cry1Ac [94]. As detailed below, Cry1Ac binds to the cadherin protein *PgCad1* in the midgut of susceptible pink bollworm larvae and resistance to Cry1Ac is associated with mutations disrupting this protein in AZP-R [96] and other strains of pink bollworm. Ocelotl et al. (2015) [94] hypothesized that Cry1Ac was not highly toxic to AZP-R because it binds to one or more midgut proteins other than PgCad1, which does not trigger the primary toxic pathway of Cry1Ac.

### 4.2. Cadherin Mutations Associated with Pink Bollworm Resistance to Cry1Ac

**Figure 3:**
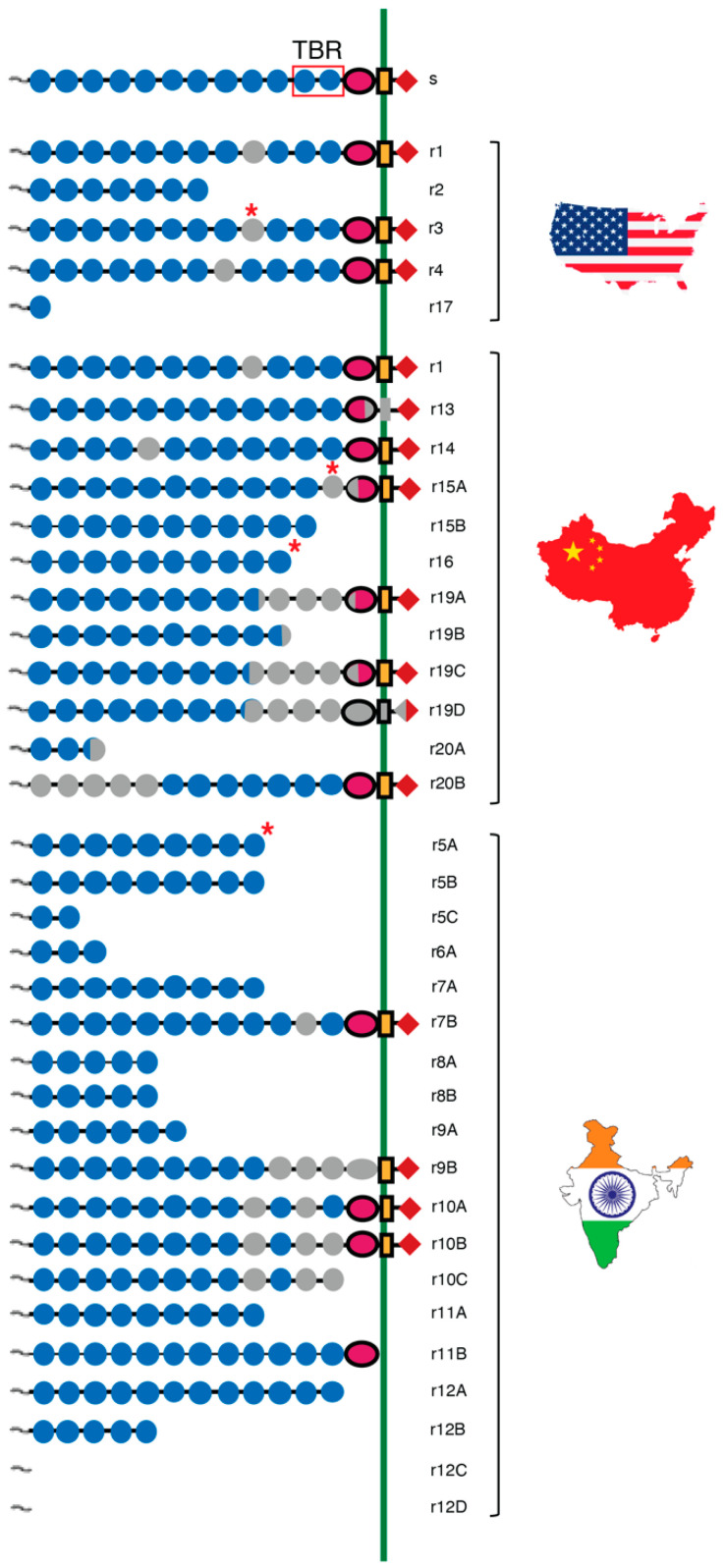
Cadherin protein variants encoded by wild-type (*s*) and 19 mutant resistance alleles (*r1*–*r17* and *r19*–*r20*) of the pink bollworm gene *PgCad1* from the U.S., China, and India. Letters *A*–*D* following the numbers for resistance alleles indicate variation in cDNA for a given gDNA sequence (e.g., *r15A* and *r15B*). The wild-type protein consists of the amino-terminal membrane signal sequence (~), cadherin repeats 1–12 (blue circles), membrane proximal region (purple oval), transmembrane region (orange rectangle), and cytoplasmic domain (red diamond). The open red box in the wild-type protein indicates the primary Cry1Ac toxin-binding region (TBR) in CR11 and CR12. Truncated structures show protein variants predicted from cDNA with premature stop codons. Gray in circles and ovals indicates missing regions caused by deletions or mis-splicing of pre-mRNA. The green vertical line represents the membrane of midgut epithelial cells with extracellular space to the left and cytoplasm to the right. Asterisks indicate transposon insertion sites (see Section 4.2.3).

Resistance to Cry1Ac and Cry1Ac-producing Bt cotton is associated with mutations in the pink bollworm cadherin gene *PgCad1* in lab-selected strains from China and the U.S. as well as field-selected populations from India [51,62,84,96,98,99,100,101,104,108,109,119,120,121]. The wild-type PgCad1 protein has 1735 amino acids [96] that form four domains: a cytoplasmic domain, a transmembrane domain, and two extracellular domains (Figure 3). The two extracellular domains consist of the membrane proximal region (MPR) and 12 cadherin repeats (CR1-CR12) (Figure 3). Eleven cadherin repeats were previously reported for PgCad1 [84,96], but updated software identifies 12 cadherin repeats [99]. Cry1Ac protoxin and activated toxin bind to recombinant peptides corresponding to wild-type CR9-CR10, CR11, CR12, and MPR but not to CR7 or CR8, which are farther from the MPR [84]. Although 20 mutant *PgCad1* alleles associated with resistance to Cry1Ac (*r1*–*r20*) have been reported, our analyses here do not include *r18* from China [62] because its sequence has not been published yet.

#### 4.2.1. PgCad1 Resistance Alleles Differ between Lab- and Field-Selected Pink Bollworm

None of the 11 published *PgCad1* resistance alleles identified from lab-selected strains from the U.S. (*r1*–*r4* and *r17*) or China (*r13*–*r16* and *r19*–*r20*) were found in field-selected populations from India, which are the source of eight other resistance alleles (*r5*–*r12*) (Figure 3, Table S1). Alleles *r1* and *r2* were first identified in a lab-selected strain from the U.S. [96] then later found in China [61,62]. Each of the other 17 resistance alleles have been found in only one of the three countries.

**Figure 4:**
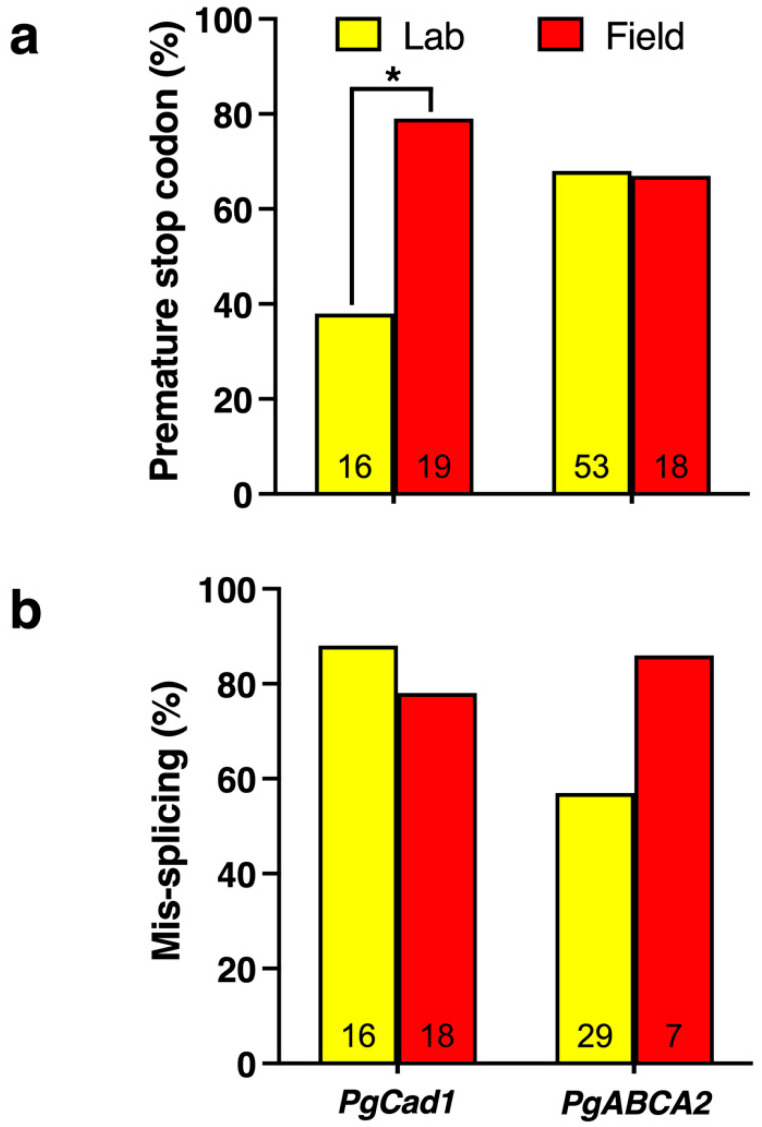
Percentage of mutations associated with pink bollworm resistance to Bt toxins involving (**a**) premature stop codons or (**b**) mis-splicing of pre-mRNA. Results from field-selected populations from India (red) or lab-selected strains (yellow) from the U.S. and China (PgCad1/Cry1Ac resistance) or only from the U.S. (*PgABCA2*/Cry2Ab resistance). The asterisks indicate a significant difference between lab- and field-selected pink bollworm in the percentage of *PgCad1* mutations that involve a premature stop codon (Fisher’s exact test, *p* = 0.02). No significant difference between lab- and field-selected pink bollworm occurs in the other three analogous pairwise comparisons shown (Fisher’s exact test, *p* > 0.20 in each comparison). The numbers at the bottom of each bar are the total number of mutations evaluated for that bar. Data from Tables S1 and S2.

The percentage of *PgCad1* mutations introducing premature stop codons is higher for India (79%, 15 of 19) than for the U.S. and China (38%, 6 of 16) (Fisher’s exact test, *p* = 0.02, Figure 4, Table S1). Overall, 80% of the mutations introducing premature stop codons were associated with the mis-splicing of pre-mRNA (Table S1). However, the percentage of mutant *PgCad1* transcripts associated with mis-splicing was similar for India (78%, 14 of 18) compared with the U.S. and China (88%, 14 of 16, Fisher’s exact test, *p* = 0.66, Figure 4, Table S1).

The expected effects of the mutations on the PgCad1 protein include loss of 5 to 1706 amino acids (Table S1). Fourteen of the *PgCad1* resistance alleles have mutations that cause either truncation or loss of the primary Cry1Ac toxin-binding region (CR11–CR12): two from the U.S. (*r2* and *r17*), four from China (*r15*, *r16*, *r19*, and *r20*), and all eight from India (*r5–r12*) (Figure 4). The mutations in the other five resistance alleles do not encode altered amino acids in the Cry1Ac toxin-binding region but may reduce toxicity by altering amino acids in other regions and changing the three-dimensional structure of the protein, thereby interfering with binding or other steps in the toxic pathway.

A meta-analysis of results from five lab-selected strains of pink bollworm from the U.S. revealed that among the six genotypes with two resistance alleles from *r1*–*r3*, survival on Bt cotton relative to non-Bt cotton was highest for *r1r2* larvae and lowest for *r2r3* [51], which would tend to favor *r1*. Conversely, results from multi-generational experiments on a non-Bt diet suggest that fitness costs might be higher for *r1* than *r2* or *r3* [52].

#### 4.2.2. PgCad1 Resistance Alleles in Field Populations in the U.S., China, and India

Allele-specific PCR screening of DNA for *r1, r2,* and *r3* from the U.S. detected none of these three alleles in 425 pink bollworm from India, including at least 84 that had field-selected resistance to Cry1Ac [121]. Furthermore, DNA sequencing of eight putatively Cry1Ac-resistant larvae from two field-selected populations in India revealed seven of the larvae harbored at least one severely disrupted *PgCad1* allele (*r5–r12*) [121]. No severely disruptive *PgCad1* mutations occurred in one of the putatively Cry1Ac-resistant larvae and three susceptible larvae from India [121]. The putatively resistant larva lacking disruptive *PgCad1* mutations might have had resistance conferred by mutations at other loci, or it was misidentified and was actually susceptible to Cry1Ac [121].

Although cadherin resistance alleles *r1*–*r3* were identified from lab-selected strains derived in 1997 from field populations in Arizona [96], none of these alleles were detected in PCR-based DNA screening of more than 9000 pink bollworm collected from the field in Arizona and neighboring southwestern states during 2001 to 2011 [48,49,122]. The most likely explanation is that these alleles were extremely rare in the field, which is consistent with the high field efficacy of Bt cotton producing Cry1Ac and sustained susceptibility to Cry1Ac in bioassays [49].

Wang et al. (2020) [61] used PCR to screen DNA from 19,748 pink bollworm collected from field populations in the Yangtze River Valley of China from 2011 to 2015. They tested for seven *PgCad1* resistance alleles previously identified from lab-selected strains: *r1*–*r3* from the U.S. and *r13–r16* from China. Remarkably, the most common of these seven alleles was *r1*, which accounted for 71% of the 194 resistance alleles detected. Phylogenetic analysis suggests that *r1* did not arise independently in the U.S. and China [61]. The *r2* allele was found only in a single individual, *r3* was not detected, and *r13–r16* accounted for the remaining resistance alleles. The frequency of all resistance alleles pooled decreased 2.3-fold from 0.0105 (95% CI: 0.0084–0.0132) in 2012 to 0.0046 (0.0031–0.0067) in 2015. Consistent with results showing none of the 4320 field-derived individuals tested in bioassays from 2011 to 2015 were resistant to Cry1Ac [44], the frequency for all resistance alleles pooled from 2012 to 2015 was 0.0049 [61], which yields an expected frequency of less than one in 40,000 homozygous resistant individuals (0.000024).

Wang et al. (2022) [62] used an F_2_ screen to test 145 single-pair lines derived from crossing susceptible females with males collected from the field in the Yangtze River Valley in 2017. In principle, this approach could identify alleles at any genetic locus conferring resistance to Cry1Ac. However, each of the seven resistant lines established had one *PgCad1* resistance allele: two lines had *r1*, two had *r13,* and one had *r15* (all previously found in China), whereas two had one novel allele each (*r19* or *r20*). This does not rule out mutations at other loci conferring resistance to Cry1Ac, but it does imply that such mutations were less common than the cadherin mutations. It is also striking that in the F_2_ screen, as in the PCR study by Wang et al. (2020) [61], no *PgCad1* resistance allele was more common than *r1*.

The frequency of the *r1* allele did not differ significantly between 2012–2015 (0.0035, based on PCR) [61] and 2017 (0.0069 based on the F_2_ screen) [62] (Fisher’s exact test, *p* = 0.28). However, excluding the novel alleles *r19* and *r20*, which could not have been detected by PCR in the 2012–2015 study, the frequency of all *PgCad1* resistance alleles pooled was 3.5 times higher in 2017 (0.017) [62] than in 2012–2015 (0.0049) (Fisher’s exact test, *p* = 0.02). In addition, the mean resistance ratio for Cry1Ac was 2.2 (SE = 0.1) for three strains derived from field populations in the Yangtze River Valley in 2017, which is significantly greater than one (one-sample *t*-test, df = 2, *t* = 15.5, *p* = 0.004). Nonetheless, none of the 72 larvae tested from each of the three field-derived strains survived exposure to a diagnostic concentration of Cry1Ac (10 micrograms Cry1Ac per ml diet) [62]. Overall, the data show resistance to Cry1Ac remained rare in the Yangtze River Valley, but close monitoring is warranted based on the small but significant increases in resistance allele frequency and LC_50_ detected in 2017.

#### 4.2.3. PgCad1 Mutations Caused by Transposable Elements

The mobilization of transposable elements can introduce mutations that confer resistance to insecticides and plant xenobiotics [123,124]. Transposon insertion into *PgCad1* is associated with resistance to Cry1Ac in at least four pink bollworm resistance alleles: one from the U.S. (*r3*), one from India (*r5*), and two from China (*r15* and *r16*) [100,101,120,121]. For *r3*, a 4739-bp insert corresponding to the active chicken repeat retrotransposon named *CR1-1_Pg* is inserted into exon 21 of *PgCad1* [120]. This insertion causes the mis-splicing of pre-mRNA and the complete loss of *PgCad1* exon 21 [120]. The *CR1-1_Pg* element also disrupts an intronic sense long non-coding RNA (lncRNA) produced from intron 20 that positively regulates *PgCad1* transcription [125]. The *r5* allele from India harbors an insertion that shares sequence similarity with several transposable elements [121]. A 3370-bp insertion corresponding to the *r15* allele consists of the remnants of three different transposable elements: *MITE1_PGo* (a miniature inverted-repeat transposable element), *RTE-5_PGo* (a retrotransposable element type 5), and *SINE-1_PGo* [101]. All three elements are inactivated but nested within each other in exon 28. *MITE1_PGo* is inserted in exon 28, *RTE-5_PGo* is inserted into *MITE1_PGo*, and *SINE-1_PGo* is inserted into *RTE-5_PGo*. [101]. The *r16* allele harbors a 1545-bp insertion in exon 20 and shares sequence similarity with the Penelope non-LTR transposon T2 [100]. The insertion leads to mis-splicing at the exon/intron 20 splice junction and introduces a premature stop codon [100].

#### 4.2.4. Localization of PgCad1 on the Cell Membrane

In addition to alterations of PgCad1 that may directly block its binding of Cry1Ac, mutations in *PgCad1* that interfere with cellular trafficking and thereby reduce the amount of this protein available for binding on the midgut membrane might also cause resistance [99,100,101]. Wang et al. (2019) [100] found that in fourth instar larvae, localization of PgCad1 protein on the apical membrane occurred with wild-type PgCad1 from a susceptible strain but not with mutant PgCad1 protein encoded by *r16* from a resistant strain (AQ65). Furthermore, in separate experiments using cultured *Hi5* insect cells to produce recombinant proteins, wild-type PgCad1 protein was transported to the cell membrane, whereas mutant PgCad1 proteins encoded by *r13*, *r15*, and *r16* were retained in the endoplasmic reticulum [99,100,101].

#### 4.2.5. Reduced Expression of PgCad1

Relative to a susceptible strain (APHIS-S), the abundance of *PgCad1* mRNA was reduced in two lab-selected resistant strains from the U.S.: by 190-fold in APHIS-R and 2.8-fold in AZP-R [98]. APHIS-R, derived from APHIS-S by selection with Cry1Ac, had greater than 500-fold resistance to Cry1Ac relative to APHIS-S [98]. Although the *r17* allele containing a premature stop codon was identified in APHIS-R, the predominant *PgCad1* transcript in this strain is full length and 99.8% identical to the wild-type sequence from APHIS-S [98]. Because no consistent differences occurred between APHIS-R and APHIS-S in the sequence of the *PgCad1* promoter, the reduced transcription of *PgCad1* is apparently caused by one or more trans-acting regulatory factors [98]. AZP-R was started by pooling insects collected from 10 Arizona cotton fields [126]. After repeated lab selection with Cry1Ac, AZP-R had up to 3100-fold resistance to Cry1Ac, which is genetically linked with *PgCad1* resistance alleles (*r1*, *r2*, and *r3* initially, *r2* was the predominant allele when mRNA abundance was measured) [52,96,115].

The results from APHIS-R and AZP-R suggest that both quantitative and qualitative changes in PgCad1 may contribute to resistance within single strains of pink bollworm [98]. Some *PgCad1* resistance alleles harboring premature stop codons (e.g., *r2* and *r17*) might lead to a reduction in transcript abundance by nonsense-mediated decay, which involves the degradation of aberrant mRNAs [127,128]. Additional studies are needed to better understand the regulation of the *PgCad1* gene in Cry1Ac-resistant pink bollworm.

## 5. Pink Bollworm Resistance to Cry2Ab

### 5.1. Modes of Resistance to Cry2Ab in India and the U.S.

Despite fewer studies of pink bollworm resistance to Cry2Ab than Cry1Ac, the mode of resistance appears to be more diverse for Cry2Ab (Table 1). Results from experiments with live insects evaluating the resistance ratio and mode of inheritance of pink bollworm resistance to Cry2Ab have been reported for four independent strains: Bt4-R2 and BX-R (derived from BX-R1 and BX-R2) from the U.S., which were each selected with Cry2Ab only in the lab [115,116,129]; CRISPR-R2, which was started by CRISPR/Cas9 gene editing of the susceptible APHIS-S strain from the U.S. then selected with Cry2Ab in the lab (see details below) [130]; and PFR from India, which was derived from field-selected populations then selected with Cry2Ab in the lab [113]. The resistance ratio for Cry2Ab was 150,000 for Bt4-R2 [116], 210 for BX-R [16], and 38 for PFR [113]. The LC_50_ of Cry2Ab was not measured for CRISPR-R2, but larval survival in bioassays with 3 micrograms Cry2Ab per ml diet was 96% for CRISPR-R2 versus 0% for APHIS-S, indicating a high level of resistance [130].

Inheritance of resistance to Cry2Ab was autosomal for Bt4-R2, CRISPR-R2, and PFR but not consistently for BX-R [113,116,129,130]. Inheritance was recessive for Bt4-R2 and CRISPR-R2 but not for PFR (*h* = 0.69–0.79) and not consistently for BX-R [113,116,129,130]. As noted above for Jalagon-R and Cry1Ac, the values of *h* indicating nonrecessive resistance of PFR to Cry2Ab might be overestimated because they are based on LC_50_ values from bioassays of 5-day-old larvae rather than neonates [113]. In initial evaluations conducted with BX-R, resistance was autosomal and recessive (*h* = 0) at 10 micrograms Cry2Ab per ml diet [115]. However, after many additional selections with Cry2Ab and a total of more than 100 generations later, resistance to Cry2Ab was no longer completely autosomal or recessive for BX-R (mean *h* = 0.35 at 10 micrograms Cry2Ab per ml diet), whereas it was autosomal and recessive for Bt4-R2 tested simultaneously [129]. Fabrick et al. (2020) [129] hypothesized that in BX-R, the frequency of one or more mutations conferring nonautosomal nonrecessive resistance increased during the time between the initial and subsequent experiments.

Although selection with Cry1Ac did not cause strong cross-resistance to Cry2Ab as noted above, selection with Cry2Ab caused up to 190-fold cross-resistance to Cry1Ac in BX-R1, which is one of the two strains from which BX-R was derived [115]. By contrast, selection with Cry2Ab did not increase resistance to Cry1Ac in Bt4-R2 [116].

To survive on Bt cotton plants producing Cry1Ac + Cry2Ab, pink bollworm larvae must be highly resistant to both toxins. No larvae from BX-R1 survived on Bt cotton bolls producing Cry1Ac + Cry2Ab despite their high survival on bolls producing Cry1Ac alone and their resistance ratios of 590–600 for Cry1Ac and 27–99 for Cry2Ab in diet bioassays [115]. Thus, it appears the Cry2Ab in bolls killed BX-R1 larvae, but the Cry1Ac did not [115]. By contrast, survival on Cry1Ac + Cry2Ab cotton relative to non-Bt cotton was 0.17 for the AZP-R2 strain, which had resistance ratios of 1100 for Cry1Ac and >710 for Cry2Ab [42], with resistance to Cry1Ac derived primarily from AZP-R and to Cry2Ab from Bt4-R2 [116]. These results suggest that the ability to survive exposure to Cry2Ab in Bt cotton for AZP-R2 but not BX-R1 primarily reflects the higher resistance to Cry2Ab in AZP-R2. In light of these results, the practical implications of moderate resistance to Cry2Ab, as seen in BX-R1 and PFR, remain to be determined. Likewise, we do not know the implications of the less than 10-fold resistance to Cry2Ab reportedly correlated with alkaline phosphatase activity in some strains of pink bollworm from India [131].

### 5.2. ABC Transporter Mutations Associated with Pink Bollworm Resistance to Cry2Ab

**Figure 5:**
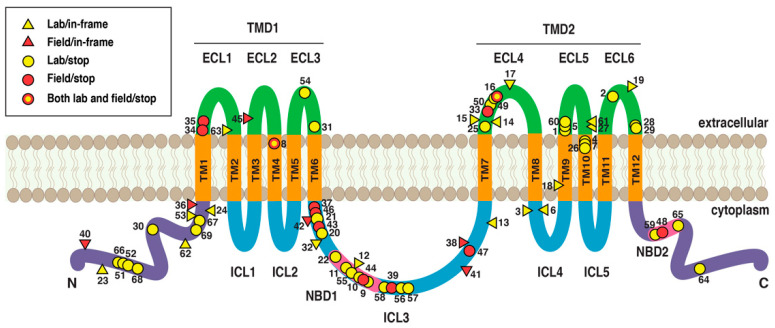
Sites affected in the PgABCA2 protein by 69 cDNA mutations associated with Cry2Ab resistance in 33 lab-selected pink bollworm from the U.S. and 8 field-selected pink bollworm from India (Table S2). The protein includes amino (N) and carboxyl (C) termini (purple), twelve transmembrane domains (TM1–TM12, orange), six extracellular loops (ECL1–ECL6, green), and five intracellular loops (ICL1–ICL5, blue). The mutations shown introduce premature stop codons (circles) or are in-frame mutations (triangles) from field-selected individuals from India (red) or lab-selected individuals from the U.S. (yellow).

Mutations disrupting the pink bollworm ATP-binding cassette (ABC) transporter gene *PgABCA2* are associated with resistance to Cry2Ab in field-selected populations from India and all three lab-selected strains from the U.S. [27,129]. As far as we know, this association has not been evaluated for the PFR strain from India. The wild-type PgABCA2 protein has 1729 amino acids [27]. Like other ABC transporters in subfamily A, its predicted structure includes two transmembrane domains, each consisting of six transmembrane regions, three extracellular loops, and two intracellular loops, with each domain connected by an intracellular loop (ICL3) (Figure 5). PgABCA2 is predicted to contain two nucleotide-binding domains: one in ICL3 and one within the intracellular cytoplasmic region (Figure 5).

Results with Bt4-R2 demonstrated a genetic linkage between *PgABCA2* and resistance to Cry2Ab [27]. In addition, introducing mutations in *PgABCA2* via CRISPR/Cas9 gene editing caused resistance to Cry2Ab in the CRISPR-R2 strain derived from the susceptible APHIS-S strain [130]. Disruptive mutations in *PgABCA2* cDNA occurred in all eight putatively resistant larvae collected from cotton plants producing Cry1Ac + Cry2Ab in four states of India during 2015–2016, but not in susceptible larvae collected from non-Bt cotton plants in India during 2010, five years before resistance to Cry2Ab was detected in India [27] (Table S2).

In contrast with *PgCad1*, where one disruptive mutation occurs within each resistance allele, many of the cDNA sequences of single *PgABCA2* clones from individual pink bollworm have more than one disruptive mutation (Table S2). Thus, we generally refer to mutations in *PgABCA2* rather than alleles. We identified 69 different mutations throughout *PgABCA2* cDNA associated with resistance to Cry2Ab (Figure 5; Table S2). The percentage of mutations introducing stop codons was nearly identical for the U.S. (68%) and India (67%) (Figure 4, Table S2).

The number of different mutations identified in *PgABCA2* cDNA is 53 in 33 Cry2Ab-resistant individuals (1.6 per individual) from the three U.S. lab strains and 18 in 8 Cry2Ab-resistant individuals (2.3 per individual) from five field-selected populations from India [27,129,130] (Figure 5; Table S2). Whereas 67 of the 69 mutations occurred in pink bollworm from either the U.S. or India, two mutations (#8 and #16) occurred in both countries (Figure 5; Table S2).

Mutation #8 was the most common mutation in Cry2Ab-resistant pink bollworm from both the U.S. and India (Table S2). It occurred in 10 of 31 (32%) individuals from the U.S., including at least one individual from all three lab strains. It also occurred in six of eight (75%) individuals from field-selected populations in India, including at least one individual from each of the four states sampled [27]. This mutation is a deletion of 145 bp (1090 to 1234) that completely skips exon 6. It causes a frameshift and introduces a premature stop codon at amino acid 373 that truncates the encoded ABCA2 protein in transmembrane region 4 (TM4, Figure 4; Table S2) [27]. In seven Cry2Ab-resistant larvae analyzed with this cDNA mutation (three from the U.S and four from India), no changes occurred in the corresponding gDNA at or near the boundaries of exon 6, which implicates mis-splicing of pre-mRNA as the underlying mechanism for this mutation [27].

Mutation #16 occurred in five Cry2Ab-resistant larvae from two of the U.S. strains and in one from India (Table S2). This mutation is a deletion of 4 bp (3097 to 3100) that causes a frameshift and introduces a premature stop codon at amino acid 1046 that truncates the encoded protein at extracellular loop 4 (ECL4, Figure 5; Table S2). The mechanism underlying this mutation is the mis-splicing of exon 18 (Table S2) [129]. For the mutations where data are available to evaluate mis-splicing (including #8 and #16), the percentage of mutations involving mis-splicing does not differ significantly between the U.S. (59%, 17 of 29) and India (86%, 6 of 7) (Fisher’s exact test, *p =* 0.23, Figure 4).

### 5.3. PgABCA2 Mutations Introduced by CRISPR/Cas9 Gene Editing

As noted above, the Cry2Ab-resistant pink bollworm strain CRISPR-R2 was generated from the susceptible APHIS-S strain from the U.S. by CRISPR/Cas9 knockout of *PgABCA2* [130]. In 11 Cry2Ab-resistant individuals from CRISPR-R2 or from F_2_ progeny between CRISPR-R2 x APHIS-S crosses, 17 different disruptive mutations in *PgABCA2* gDNA and 26 in *PgABCA2* cDNA were identified [130] (Table S2). Among the 26 different cDNA mutations, 22 introduced premature stop codons, and four had in-frame deletions causing loss of 42 to 1442 amino acids [130] (Figure 4; Table S2). Although the 17 gDNA mutations were probably caused by gene editing, only 9 of the 26 cDNA mutations occurred precisely within a *PgABCA2* single guide RNA (sgRNA) target site and six affected exons containing an sgRNA site [130]. For the remaining 11 cDNA mutations outside the sgRNA sites, five were previously identified either from Cry2Ab lab-selected strains from Arizona (i.e., Bt4-R2 and/or BX-R), from field-selected populations from India, or both [130] (Table S2). The mutations outside the sgRNA sites could reflect Cas9 acting outside the target sites, existing genetic variation in APHIS-S, or both. Because alleles conferring resistance to Cry2Ab were rare in APHIS-S [16,114,115,116,129,130], it seems most likely that these mutations were caused by off-target Cas9 cleavage.

### 5.4. An Alternative Mechanism of Resistance to Cry2Ab in the BX-R Strain from the U.S.

Although mutations in *PgABCA2* occurred in the lab-selected BX-R strain, some evidence also implies that mutations in at least one other locus contributed to Cry2Ab resistance in this strain [129]. Survival at a diagnostic concentration of Cry2Ab (3 μg Cry2Ab per mL diet) was 100% for BX-R and Bt4-R2 [129]. If resistance was conferred entirely by mutations at the same locus in both strains, 100% survival at this concentration would also be expected in the F_1_ progeny from crosses between the strains because all the progeny would lack alleles for susceptibility at that locus. Thus, the striking heterogeneity in responses to Cry2Ab among 20 F_1_ families from crosses between BX-R and Bt4-R2 revealed that resistance was affected by genetic variation within the strains, between the strains, or both [129].

A total of 6 of the 20 F_1_ families had survival >99%, which is consistent with a shared genetic basis of resistance between the strains for these families. Disruptive mutations in *PgABCA2* are almost certainly the shared genetic basis of resistance between the strains because of the prevalence of such mutations in BX-R, Bt4-R2, and in their F_1_ progeny that survived exposure to Cry2Ab [27,129]. However, survival of less than 70% (minimum = 24%) in 5 of the 20 families indicates that in these families, the parents from BX-R and Bt4-R2 did not share a locus where both parents had no alleles conferring susceptibility. BX-R probably harbored the mutations at an additional locus or loci contributing to Cry2Ab resistance because disruptive *PgABCA2* mutation #1 (also known as *r_A1_*) was fixed in Bt4-R2, and Cry2Ab resistance was autosomal and recessive in Bt4-R2 but not BX-R [129]. The putative additional resistance locus or loci remain to be identified.

## 6. Conclusions

The genes harboring mutations associated with resistance were similar between lab- and field-selected pink bollworm for each of the Bt toxins evaluated here, Cry1Ac and Cry2Ab. Pink bollworm resistance to Cry1Ac was consistently associated with mutations disrupting the midgut cadherin protein PgCad1 in lab-selected strains from the U.S. and China, as well as in field-selected populations from India. Similarly, in lab-selected strains from the U.S. and field-selected populations from India, resistance to Cry2Ab was usually associated with mutations in the ABC transporter protein PgABCA2. Knocking out PgABCA2 with CRISPR/Cas9 demonstrated that such mutations can cause pink bollworm resistance to Cry2Ab. Nonetheless, we cannot exclude contributions by other genes, and at least one additional locus apparently contributed to resistance to Cry2Ab in a lab-selected strain of pink bollworm.

Many different mutations are associated with pink bollworm resistance to Cry1Ac and Cry2Ab, including at least 19 in *PgCad1* and 69 in *PgABCA2* identified from several lab-selected strains and small samples from field-selected populations in India (n = 8 resistant individuals from India sequenced for each gene). We expect that expanding the sample sizes for field-selected populations would increase the number of resistance-associated mutations discovered.

Despite extensive variation in the resistance-associated mutations in *PgCad1* and *PgABCA2*, the first Cry1Ac resistance allele identified in the U.S. (*r1*) is also the most common resistance allele found in China. In addition, a second resistance allele (*r2*) was detected in both countries. However, none of the 11 *PgCad1* resistance alleles reported from lab-selected strains from the U.S. and China were detected in field-selected populations from India.

Conversely, two of the mutations in *PgABCA2* identified from lab-selected strains from the U.S. (#8 and #16) also occurred in field-selected populations from India. Moreover, mutation #8 in *PgABCA2*, a 145-bp deletion that introduces a premature stop codon, was the most common mutation in pink bollworm from the U.S. and India. These results suggest mutation #8 is especially favorable, potentially conferring a high level of resistance to cotton producing Cry2Ab, low fitness cost on non-Bt cotton, or both.

In the lab- and field-selected pink bollworm, most mutations associated with resistance to Cry1Ac and Cry2Ab are associated with mis-splicing of pre-mRNA (mean = 77%, range: 57% to 88%, Figure 4), and a substantial percentage introduce stop codons expected to yield truncated PgCad1 and PgABCA2 proteins (mean = 63%, range: 38 to 79%). Overall, the results imply that lab selection is useful for identifying the genes and general types of mutations that confer practical resistance of pink bollworm to Cry toxins, but the specific mutations causing practical resistance in the field are diverse and not limited to those detected via lab selection.

The practical implication of these results for monitoring pink bollworm resistance to Bt toxins is to consider all disruptive mutations in the genes identified via lab selection rather than just surveying for one or a few specific mutations. For molecular monitoring, this could entail targeted highly multiplexed PCR-based sequencing [132] or other approaches that can readily detect disruptive mutations throughout an entire gene. An alternative is F_1_ screens, where individual adults from a lab strain homozygous for resistance are mated individually to field-derived adults, and their F_1_ progeny are tested in bioassays [133,134]. This method can detect resistance in the field-derived adults caused by dominant mutations at any locus as well as by recessive mutations at the same locus conferring resistance in the lab strain [134].

We infer that management practices in the U.S. and China impeded the evolution of resistance to Cry1Ac rather than limited genetic variation for resistance in those countries. The results reviewed here show that lab selection generated many strains of pink bollworm from the U.S. and China in which resistance to Cry1Ac is associated with *PgCad1* mutations similar to those in field-selected populations from India. Likewise, the absence of field-evolved pink bollworm resistance to Cry2Ab in the U.S. can be attributed to effective resistance management rather than genetic constraints because *PgABCA2* mutations found in lab-selected strains from the U.S. were similar or even identical to those in field-selected populations from India.

In both the U.S. and China, refuges of non-Bt cotton appear to have helped to sustain the susceptibility of pink bollworm to Bt cotton [13,43,47,48]. Furthermore, the efficacy of Bt cotton was critical for the success of a multi-tactic program that eradicated pink bollworm from the cotton-growing regions of the continental U.S. [13,43,48,56]. Knowledge of the recessive inheritance of resistance to Cry1Ac and the associated *PgCad1* mutations was important for implementing PCR-based monitoring for resistance, designing the eradication program, and facilitating approval of the program by the U.S. Environmental Protection Agency [48,49,122,135].

Changes associated with pink bollworm resistance to Cry1Ac include reduced transcription of *PgCad1,* mislocalization of PgCad1 within cells, reduced binding of Cry1Ac to larval midgut membranes, and reduced oligomerization of Cry1Ac. Resistance to Cry2Ab is associated with reduced binding of Cry2Ab to larval midgut membranes in some other lepidopteran pests [136], but we are not aware of analogous data evaluating Cry2Ab binding or other steps in its toxic pathway for pink bollworm.

This review highlights some striking similarities between lab-selected resistance and field-selected practical resistance to Cry1Ac and Cry2Ab in pink bollworm. As far as we know, analogous comparisons of the molecular genetic basis of Bt resistance between lab-selected and practical resistance for the same pest are not yet possible for other species. Aside from pink bollworm, the only cases of practical resistance to a Bt crop where the molecular genetic basis of resistance is known to involve *Spodoptera frugiperda* resistance to Cry1Fa in Puerto Rico, the continental United States, and Brazil associated with mutations in an ABC transporter gene (*SfABCC2*) [18]. We are not aware of reports of the genetic basis of lab-selected resistance of this pest to Cry1Fa. *Helicoverpa armigera*, a widespread pest of cotton and other crops, has been selected in the lab for resistance to Cry1Ac conferred by mutations that affect cadherin (HaCad1), ABC transporters (HaABCC2 and HaABCC3), or tetraspanin (HaTSPAN1) [17,23]. We are not aware of any cases of practical resistance of this pest to Cry1Ac [14]. However, in field populations of *H. armigera* in China exposed to Bt cotton producing Cry1Ac, the dominant point mutation in *HaTSPAN1* identified in a lab-selected strain increased 100-fold from 0.001 in 2006 to 0.10 in 2016, yielding an early warning of resistance [14,17]. As more data become available, it will be intriguing to compare the molecular genetic basis of lab-selected resistance and field-selected practical resistance to Bt crops in more species of pests.

## Figures and Tables

**Table 1:** Summary of the mode of resistance to Bt toxins Cry1Ac and Cry2Ab in pink bollworm from China, India, and the United States (see text for details and references).

Resistance Trait	Country		
	China ^a^	India ^b^	U.S. ^a^
*Cry1Ac*			
Resistance ratio > 100 ^c^	Yes	Yes	Yes
Recessive inheritance	Yes	Yes	Yes
Weak cross-resistance ^d^	Yes	Yes	Yes
Reduced binding ^e^	Yes	Yes	Yes ^f^
*PgCad1* resistance alleles	*r1, r2, r13–r16, r18–r20*	*r5–r12*	*r1–r4, r17*
*Cry2Ab*			
Resistance ratio > 100 ^c^	NA ^g^	Varies ^h^	Yes
Recessive inheritance	NA	NA	Varies
Weak cross-resistance ^i^	NA	NA	Varies
Reduced binding ^e^	NA	NA	NA
*PgABCA2* mutations ^j^	NA	Yes	Varies

^a^ Lab-selected resistance; ^b^ Field-selected resistance, some strains from field-selected populations were selected further in the lab; ^c^ LC_50_ more than 100 times greater for the resistant strain than a susceptible strain; ^d^ Cross-resistance to Cry2Ab <3-fold; ^e^ Reduced binding of toxin to larval midgut membranes; ^f^ Reduced binding of Cry1Ab, but not Cry1Ac; ^g^ NA, data not available; ^h^ Resistance ratio was 38 for PFR strain, is expected to be >100 for field-selected populations that survive on Bt cotton producing Cry2Ab; ^i^ Cross-resistance to Cry1Ac <10-fold; ^j^ Two mutations (#8 and #16) occurred in both India and the U.S.; 67 others occurred in only one of the two countries.

## Data Availability

Not applicable.

## Supplementary Materials

The following supporting information can be downloaded at: https://www.mdpi.com/article/10.3390/insects14020201/s1, Table S1: *PgCad1* mutations associated with Cry1Ac resistance in pink bollworm from China, India, and the U.S.; Table S2: *PgABCA2* mutations associated with Cry2Ab resistance in pink bollworm from the U.S. and India.
