# Supplementary material for: Molecular Genetic Basis of Lab- and Field-Selected Bt Resistance in Pink Bollworm
Source: Insects. 2023 Feb 17;14(2):201. doi: 10.3390/insects14020201 (PMC9959750; doi:10.3390/insects14020201)
Supplement: Supplementary file 1 [file insects-14-00201-s001.zip › insects-2186377-supplementary/insects-2186377-proofed-supplementary/Front Page for Supplementary Information.pdf]

Supporting Information for

Molecular Genetic Basis of Lab- and Field-Selected Bt Resistance in Pink Bollworm

Jeffrey A. Fabrick [1,*], Xianchun Li [2], Yves Carrière [2], and Bruce E. Tabashnik [2]

[1] USDA ARS, U.S. Arid Land Agricultural Research Center, Maricopa, AZ 85138 USA
[2] Department of Entomology, University of Arizona, Tucson, AZ 85721 USA

[*] Corresponding author: jeff.fabrick@usda.gov

SUPPLEMENTARY INFORMATION includes:

Supplementary Table S1. *PgCad1* mutations associated with Cry1Ac resistance in pink bollworm from China, India, and the U.S.

Supplementary Table S2. *PgABCA2* mutations associated with Cry2Ab resistance in pink bollworm from the U.S. and India.
